# Exploring the dynamic interplay between cancer stem cells and the tumor microenvironment: implications for novel therapeutic strategies

**DOI:** 10.1186/s12967-023-04575-9

**Published:** 2023-10-02

**Authors:** Yan-Ruide Li, Ying Fang, Zibai Lyu, Yichen Zhu, Lili Yang

**Affiliations:** 1 Department of Microbiology, Immunology and Molecular Genetics, University of California, Los Angeles, Los Angeles, CA 90095 USA; 2 Eli and Edythe Broad Center of Regenerative Medicine and Stem Cell Research, University of California, Los Angeles, Los Angeles, CA 90095 USA; 3 Jonsson Comprehensive Cancer Center, David Geffen School of Medicine, University of California, Los Angeles, Los Angeles, CA 90095 USA; 4 Molecular Biology Institute, University of California, Los Angeles, Los Angeles, CA 90095 USA

**Keywords:** Cancer stem cell (CSC), Tumor microenvironment (TME), Cancer immunotherapy, Tumor-associated macrophage (TAM), Myeloid-derived suppressor cells (MDSC), Therapy resistance, Immune evasion

## Abstract

Cancer stem cells (CSCs) have emerged as key contributors to tumor initiation, growth, and metastasis. In addition, CSCs play a significant role in inducing immune evasion, thereby compromising the effectiveness of cancer treatments. The reciprocal communication between CSCs and the tumor microenvironment (TME) is observed, with the TME providing a supportive niche for CSC survival and self-renewal, while CSCs, in turn, influence the polarization and persistence of the TME, promoting an immunosuppressive state. Consequently, these interactions hinder the efficacy of current cancer therapies, necessitating the exploration of novel therapeutic approaches to modulate the TME and target CSCs. In this review, we highlight the intricate strategies employed by CSCs to evade immune surveillance and develop resistance to therapies. Furthermore, we examine the dynamic interplay between CSCs and the TME, shedding light on how this interaction impacts cancer progression. Moreover, we provide an overview of advanced therapeutic strategies that specifically target CSCs and the TME, which hold promise for future clinical and translational studies in cancer treatment.

## Introduction

Cancer stem cells (CSCs), also known as cancer-initiating cells, constitute a minor subpopulation within tumors characterized by their distinctive attributes, such as self-renewal capability and multilineage differentiation potential within the tumor microenvironment (TME) [1–3]. CSCs have been implicated in fundamental processes of tumor initiation, growth, metastasis, and acquisition of therapy resistance. These cells exhibit inherent resistance to conventional anti-cancer therapies and hostile microenvironmental conditions, contributing to disease recurrence and treatment failure [4–6]. Understanding the complex biology and behavior of CSCs is imperative for advancing therapeutic strategies targeting these resilient cells and improving patient outcomes in the context of cancer treatment.

TME encompasses non-cancerous cells, extracellular matrix components, and signaling molecules that surround the tumor [7–10]. It functions as a dynamic niche with influential capabilities over tumor cell behavior, immune responses, angiogenesis, and response to therapy [11–15]. Within the TME, numerous immunosuppressive cell populations exist, including tumor-associated macrophages (TAMs), myeloid-derived suppressor cells (MDSCs), cancer-associated fibroblasts (CAFs), and regulatory T cells (Tregs). These cell types collectively contribute to immune reactivity suppression and facilitate tumor progression [16–21]. Notably, there exists a reciprocal relationship between CSCs and the TME, wherein the TME promotes the maintenance of CSCs in a stem-like state, supporting their survival, self-renewal, and resistance to therapy through intricate cellular and molecular mechanisms [22–24]. Conversely, CSCs produce factors that drive the polarization and persistence of the TME in an immunosuppressive state [25–33]. Given these obstacles, further investigations are necessary to surmount the limitations of current immunotherapies and develop targeted approaches that address both CSCs and the TME.

Now, an increasing number of researchers have directed their attention towards modifying the TME and targeting CSCs as potential therapeutic strategies. For instance, approaches involving immune checkpoint inhibitors [34–36], CAR-T cell therapies [16, 37, 38], and nanoparticle-based drug delivery systems [39, 40] have shown promise in modulating the TME and enhancing immune responses against CSCs. Despite these advancements, several challenges persist. Firstly, the heterogeneity and plasticity of CSCs make their specific targeting and eradication a complex task [41]. Additionally, the intricate interplay between CSCs and the TME, as well as the presence of immunosuppressive factors within the TME, can impede the efficacy of therapeutic interventions [25–28]. Furthermore, the development of resistance mechanisms by CSCs and the TME remains a significant obstacle that needs to be overcome to achieve long-term treatment success. Addressing these challenges requires continued research efforts to unravel the underlying mechanisms and devise innovative strategies that can effectively alter the TME and eliminate CSCs, ultimately improving outcomes for cancer patients.

In this review, we aim to outline the recent emerging results that contribute to the definition of CSC status. We also delve into the remarkable strategies employed by CSCs, which allows them to evade immune surveillance and manipulate the immune editing process, leading to resistance against immunotherapies. Furthermore, we provide an in-depth exploration of the current state of the art regarding the features of CSC-TME interactions. Additionally, we discuss recently discovered therapeutic strategies that specifically target microenvironmental components, exerting an influence on CSC activities. Finally, we propose novel and promising CSC and TME-targeted therapeutic strategies for the advancement of robust cancer immunotherapy.

## Signature properties of cancer stem cells

### CSC characteristics and functional properties

CSCs constitute a distinct subpopulation within tumors, exhibiting notable features that contribute to tumor dynamics and therapy resistance [42]. Self-renewal and differentiation capabilities represent fundamental characteristics of CSCs. Through self-renewal, CSCs perpetuate tumor growth by generating new CSCs, while differentiation yields non-CSC tumor cells, contributing to tumorigenicity, tumor cell heterogeneity, and the hierarchical organization structure of cells in tumors [43]. Studies have revealed the capacity of CSCs to sustain their self-renewal ability through successive passages in vitro and implantations in vivo [44, 45]. Notably, another characteristic that CSCs possess is the inherent resistance to chemotherapy and radiation therapies, largely attributed to their quiescent and slow-cycling nature, which renders them less susceptible to the effects of these treatments compared to rapidly dividing non-CSC tumor cells [46]. Additionally, CSCs display specific surface antigens and markers, such as CD44 and CD133 in various cancers [47–52], as well as EpCAM in epithelial cancers [53, 54]. These markers enable the identification, isolation, characterization, and targeting of CSCs, facilitating a comprehensive understanding of their heterogeneity and therapeutic implications.

### CSC heterogeneity and plasticity

CSC plasticity, a pivotal concept in tumor biology, involves the ability of CSCs to undergo various transitions, significantly influencing tumor initiation, heterogeneity, and metastasis. CSCs exhibit the capacity for dedifferentiation and transitioning between cell states [55]. These transitions include the epithelial-mesenchymal transition (EMT), promoting invasion and metastasis, and its counterpart, mesenchymal-epithelial transition (MET), facilitating colonization at secondary sites. Furthermore, CSCs can shift between differentiated and dedifferentiated states, adopting hybrid E/M states, thus contributing to intratumoral heterogeneity and therapeutic resistance [55, 56].

With their unique characteristics, CSCs are believed to play a critical role in tumor initiation, heterogeneity and metastasis [57]. As mentioned, CSCs possess the remarkable ability to give rise to differentiated progeny within the tumor, while maintaining their self-renewal capacity, thus sustaining tumor growth. Additionally, the plasticity of CSCs plays a significant role in promoting stemness, as evidenced by the overexpression of stemness-associated transcription factors (e.g., OCT3/4, SOX2, NANOG) among others, in diverse tumor types [58, 59]. These transcription factors, in turn, exert influence on many intracellular signaling pathways, including the Wnt/TCF4 and STAT3 pathway, thereby contributing to tumor initiation and progression in response to treatments [60, 61]. Notably, a recent study has revealed that lineage plasticity in late-stage prostate cancer depends on JAK/STAT and fibroblast growth factor receptor (FGFR) inflammatory signaling [61]. Furthermore, the metastatic potential of tumors predominantly arises from the EMT undergone by CSCs, a process intricately connected to their plasticity. OCT4 expression regulates EMT-related genes (e.g., CXCR4, MMR9, MMR2, and TIMP1) further underscoring the interplay between plasticity and EMT [62]. CSCs also exhibit enhanced resistance to anoikis, a form of cell death triggered by detachment from the extracellular matrix. This resistance enables CSCs to survive within the bloodstream, facilitating their establishment as secondary tumors at distant anatomical sites [63].

Mesenchymal stem cells (MSCs) are a distinct cell population with relevance to tumor progression. These cells are known for their capacity to migrate to tumor sites and exert influences on cancer-related processes such as cell proliferation, angiogenesis, and metastasis [64]. Within the TME, MSCs are often recruited through chemotactic signals, including chemokines and cytokines originating from cancer cells [65]. MSCs can also induce EMT in cancer cells, where cancer cells acquire invasive properties and increased resistance to therapeutic interventions. Notably, there are intricate interactions and associations between CSCs and MSCs within the framework of the TME [66]. One hypothesis posits that certain CSC populations may originate from resident tissue stem cells, including MSCs. This theoretical framework suggests that genetic mutations or epigenetic alterations within MSCs or other stem cell cohorts could facilitate their transformation into CSCs [66]. It is crucial to acknowledge that the precise origins of CSCs exhibit variability contingent on the cancer type under investigation. MSCs exhibit the capacity to release various bioactive factors that can influence stemness, drug resistance, or the maintenance of the CSC phenotype [66]. For instance, in ovarian cancer, MSCs have been documented to produce BMP4 and BMP2, thereby amplifying the population of CSCs [67]. Additionally, MSCs have demonstrated the ability to modulate the metabolic profiles of CSCs by secreting exosomes, a phenomenon observed in breast cancer and cholangiosarcomas [68, 69]. In summary, the intricate interplay between MSCs and CSCs within the TME underscores the multifaceted roles of these cell types in cancer progression and highlights potential avenues for therapeutic exploration.

### Controversial models of CSC-induced tumorigenesis and heterogeneity

**Fig. 1:**
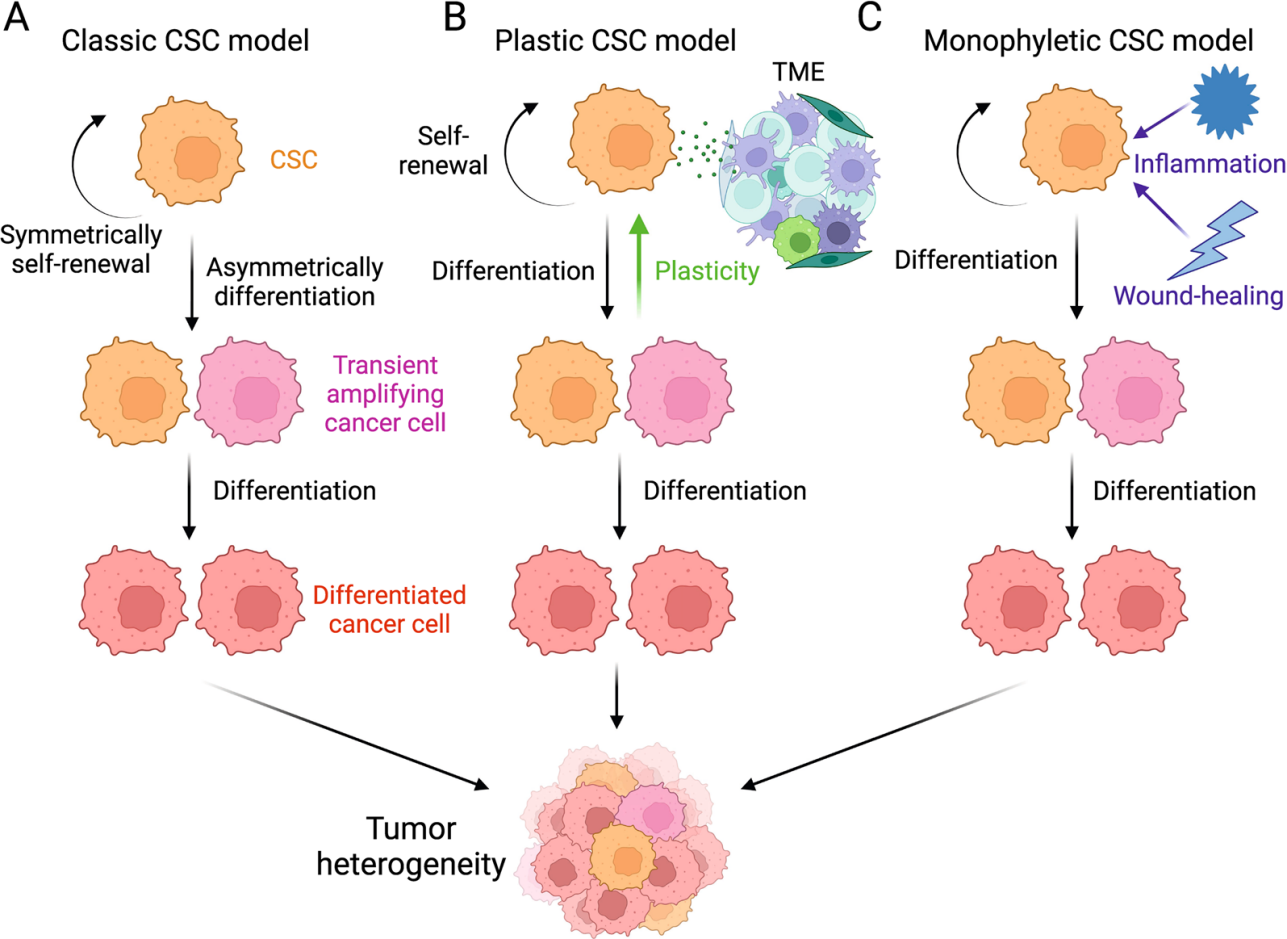
CSC-induced tumorigenesis models. **A** Classical CSC Model. CSCs exhibit asymmetric division, resulting in CSC renewal and the generation of less tumorigenic non-CSC daughter cells. **B** Plastic CSC Model. Tumor microenvironment shapes CSC plasticity, explaining bidirectional conversion between CSC and non-CSC. **C** Monophyletic CSC Model. Inflammation and wound-healing signals impact aberrant differentiation and dynamic changes in tumor pathology

Currently, the targeted therapies against CSCs encounter challenges related to specificity, heterogeneity, and plasticity, leading to limited efficacy and potential side effects. The specificity challenge is closely associated with the classical CSC model, which proposes a hierarchical tumor structure with a subset of cells (CSCs) possessing self-renewal and tumorigenic abilities [55, 70]. Therapies designed to target CSC-specific markers and pathways may be effective in eliminating CSC in this context. However, the more advanced plastic CSC model suggests bidirectional conversions, where non-CSCs continually generate CSC populations during tumorigenesis, highlights the need for treatments that can adapt to the dynamic nature of CSCs, targeting both CSCs and the tumor cells in transition. Another theory, the dualistic origin model, considers tumors arising from blastomeres or reprogrammed somatic cell-derived stem cells [71]. However, this model overlooks dynamic changes in tumor histopathology, including conversions during chemotherapy. The recent monophyletic model highlights aberrant differentiation and the influence of signaling pathways regulating inflammation and wound repair in cancer development [72]. Despite advancements, this model has limitations in neglecting the tumor microenvironment and lacking experimental evidence (Fig. 1).

These diverse models not only highlight the inherent heterogeneity within CSC populations but also underscore the fact that CSCs are far from being a homogeneous entity. This intrinsic heterogeneity presents a formidable challenge for the development of effective treatment strategies, as therapies designed based on a single model may not yield universal success. Furthermore, the concept of plasticity, as introduced by the plastic CSC model, adds an extra layer of complexity by proposing that the phenotypic characteristics of CSCs can dynamically change over time and in response to treatment [55]. This challenges the conventional notion of CSCs as a static and easily targetable population. Consequently, these factors contribute to the ongoing difficulty in definitively identifying markers for CSCs [73]. Although CSCs are often characterized using specific surface markers or cellular features, these markers can vary across different tumor types and even within individual tumors. Additionally, the dynamic nature of CSCs and their capacity to transition between states further complicates the task of marker identification. These collective challenges in delineating CSC markers underscore the pressing need for more precise and adaptable approaches in the realm of CSC research and treatment.

## Crosstalk between cancer stem cells and the tumor microenvironment

### Components of the tumor microenvironment

**Fig. 2:**
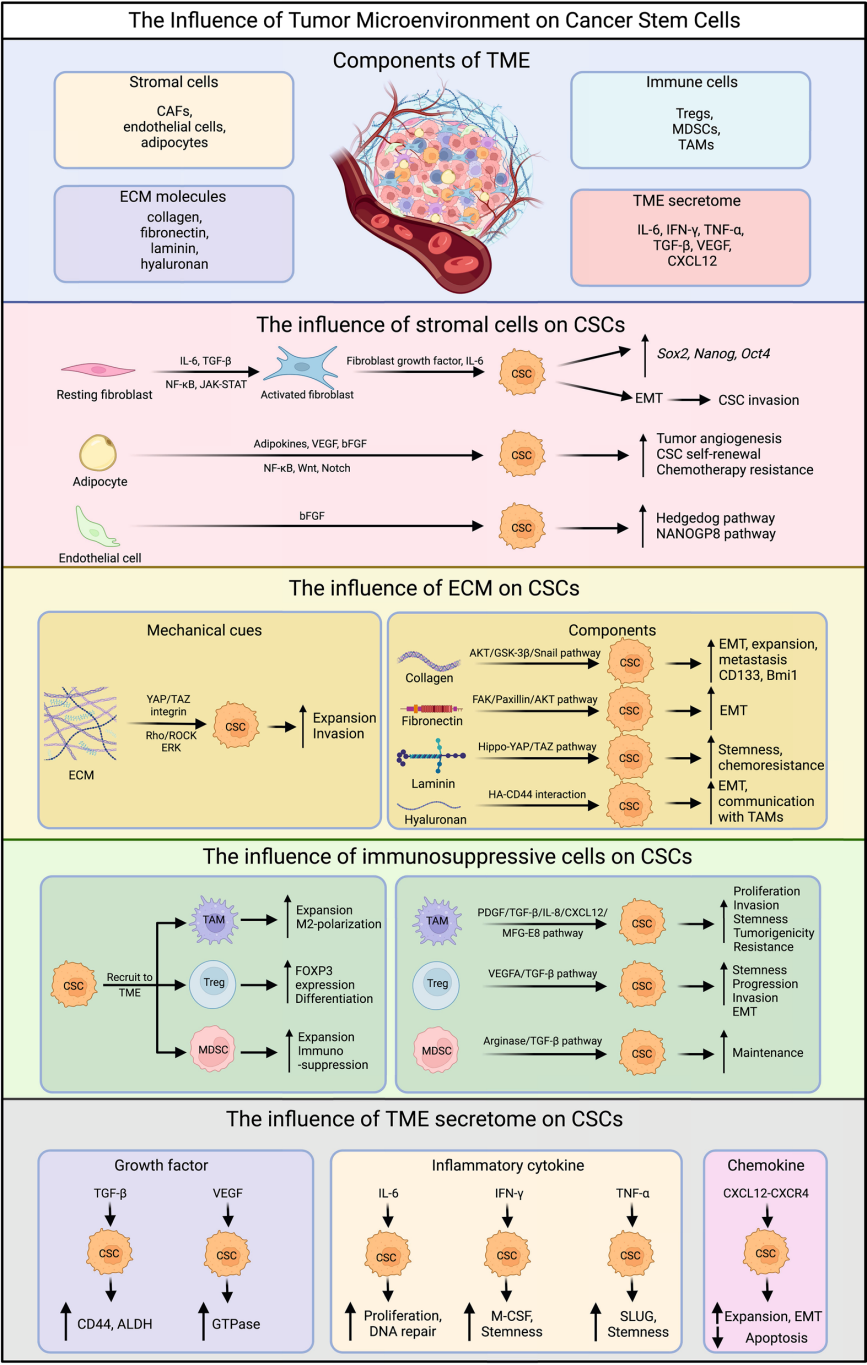
The influence of TME on CSCs. The figure shows the TME components and their influence on CSCs, including the influence of stromal cells, ECM, immunosuppressive cells, and TME secretome. CAF, cancer-associated fibroblast; Treg, regulatory T cell; MDSC, myeloid-derived suppressor cell; TAM, tumor-associated macrophage; ECM, extracellular matrix; IL-6, interleukin-6; IFN-γ, interferon-gamma; TNF-α, tumor necrosis factor-alpha; TGF-β, transforming growth factor-beta; VEGF, vascular endothelial growth factor; CXCL12, C-X-C motif chemokine ligand 12; bFGF, basic fibroblast growth factor; HA, hyaluronan; EMT, epithelial-mesenchymal transition; ALDH, aldehyde dehydrogenase; M-CSF, macrophage colony-stimulating factor; CXCR4, C-X-C chemokine receptor type 4

The TME is a complex network consisting of various cellular and non-cellular elements that play a significant role in tumor development and progression (Fig. 2). Stromal cells, including CAFs, mesenchymal stem cells (MSCs), endothelial cells, and adipocytes, are involved in maintaining TME, promoting angiogenesis, facilitating extracellular matrix (ECM) remodeling, inducing drug resistance, and accelerating extravasation and metastasis [11–15]. Within the ECM certain molecules such as collagen, fibronectin, elastin, laminin, and in some cancers, hyaluronan (hyaluronic acid or HA), have been closely associated with unfavorable prognoses and resistance to therapy [74, 75].

In the TME, both innate and adaptive immune cells display dual functions in regulating tumor activities[76]. Innate immune cells, such as TAMs and MDSCs, tend to play inhibitory roles, while the adaptive branch is mainly comprised of Tregs [16, 77]. Moreover, soluble factors like interleukins, interferons, tumor necrosis factors, growth factors, and chemokines, which are secreted by stromal cells, immune cells, and tumor cells in the TME, have the capacity to downregulate immune responses and promote tumor cell proliferation and angiogenesis [78, 79].

### Influence of the stromal cells on CSC-driven tumor progression

Various types of stromal cells play a crucial role in maintaining CSCs and promoting their functions through diverse mechanisms (Fig. 2). Signaling molecules like IL-6 and growth factors such as TGF-β, released by nearby cancer cells or immune cells, stimulate the development of CAFs by activating the NF-κB and JAK-STAT pathways [80, 81]. Activated CAFs have the capacity to support CSC self-renewal by releasing growth factors, including fibroblast growth factors, and cytokines like IL-6 [82]. This sustains the stem-like properties of CSCs and the expression of stemness-related genes such as *Sox2*, *Nanog*, and *Oct4* [58, 59]. Furthermore, CAFs contribute to the construction of the ECM, leading to the induction of EMT in cancer cells, including CSCs, and this process exacerbates CSC invasion into adjacent tissues, thereby promoting metastasis [83].

Endothelial cells, another vital component of stromal cells in the TME, play a significant role in promoting angiogenesis, which supplies nutrients required for tumor growth and supports metastasis [84]. CSCs and other tumor cells secrete pro-angiogenic factors such as vascular endothelial growth factor (VEGF) and basic fibroblast growth factors (bFGF), thereby recruiting and activating endothelial cells in the local TME [85]. Reciprocally, endothelial cells maintain CSC stem-like characteristics and enhance CSC-driven tumor growth by releasing soluble factors, including bFGF, and activating specific intracellular pathways, such as the Hedgehog pathway in glioma cancer cells and the NANOGP8 pathway in colorectal cancer cells, in different cancer types [86]. Moreover, under oxidative stress, CSCs can differentiate into tumor endothelial cells, fulfilling their nutrient and oxygen needs through pentose phosphate pathway inactivation and autophagy [87].

Adipocytes within the TME also contribute to CSC behavior and tumor progression. When stimulated by CSCs, adipocytes secrete elevated levels of adipokines and immunosuppressive cytokines, such as adiponectin, leptin, visfatin, resistin, and IL-6 [88]. Notably, elevated levels of leptin strongly correlate with tumor angiogenesis, CSC self-renewal, and chemotherapy resistance. In breast cancers, adipocytes enhance CSC properties and tumor proliferation through signaling pathways like NF-κB, Wnt, and Notch, by secreting leptin, visfatin, and resistin [88, 89].

### Influence of the ECM on CSC-driven tumor progression

The components of the ECM play a pivotal role in influencing CSC behavior through diverse mechanisms (Fig. 2). In different cancer types, various types of collagen molecules present in the surrounding ECM regulate CSC stemness [90]. For instance, Type I collagen, the most abundant collagen protein, enhances stemness-related characteristics in cancer cells, upregulates stemness-related markers like CD133 and Bmi1 in human colorectal cancers through α2β1 integrin, and provides support for the niche required for CD133^+^ glioblastoma stem cells (GSCs) maintenance and growth [91]. Similarly, Type IV collagen promotes the expansion of head and neck squamous carcinoma cancer stem cells (HNSC-CSCs), and inhibiting collagen type IV alpha 2 (COL4A2) by siRNA significantly reduces the expansion and metastasis of triple-negative breast cancer (TNBC) [92]. Additionally, collagen type XI α1 (COL11A1) enhances EMT and cancer cell stemness in pancreatic ductal adenocarcinoma (PDAC) through the AKT/GSK-3β/Snail signaling pathway [93].

Fibronectin overexpression promotes EMT and stem cell-like characteristics in ovarian carcinoma cells, leading to increased differentiation ability of GSCs through the FAK/Paxillin/AKT signaling pathway and inducing chemoresistance to drugs like carmustine [94]. Another glycoprotein, laminin-332, maintains the stemness and chemoresistance of human hepatic CSCs, while laminin-511, stimulated by breast CSCs, reciprocally supports breast cancer self-renewal through α6Bβ1 integrin and the Hippo-YAP/TAZ pathway [95]. HA, a non-proteoglycan polysaccharide, induces EMT, facilitates communication between breast CSC and TAMs, and promotes chemoresistance through HA-CD44 interaction [96].

These ECM molecules provide mechanical signals to CSCs, and different ECM compositions lead to varying ECM stiffness [97]. The mechanical cues from ECM stiffness are transduced to local CSCs through signaling pathways like YAP/TAZ, integrin, and the Rho/ROCK pathway [97]. Upon stimulation, CSC proliferation is indirectly augmented via the extracellular signal-regulated kinase (ERK) pathway [98]. Furthermore, CSC invasion and metastasis are promoted by TGF-β-induced EMT and the permeable basement membrane constructed by ECM components, particularly CAFs [99].

Recent research underscores the interconnection between the ECM and the pan-cancer significance of the Rho and Hippo/YAP pathways [100]. These pathways, intimately linked to G protein-coupled receptor (GPCR) signaling, exert profound effects on CSC within the context of the ECM. GPCR activation initiates a cascade of events, encompassing G protein activation and cytoskeletal rearrangements, which not only influence the phosphorylation of YAP and TAZ but are also intimately connected to the mechanical cues provided by the ECM [100]. In CSCs, renowned for their stemness attributes, including self-renewal and resistance to conventional therapies, GPCR-induced YAP/TAZ activation accentuates these traits. Moreover, these pathways govern critical aspects of CSC behavior, impacting tumor initiation, progression, and metastasis, all within the dynamic microenvironment orchestrated by the ECM [100].

### Influence of immunosuppressive cells on CSC-driven tumor progression

The communication between CSCs and immunosuppressive cells, including Tregs, MDSCs, and TAMs, plays a pivotal role in shaping the TME and influencing cancer progression (Fig. 2) [101]. CSCs have been shown to possess a more efficient capacity for recruiting Tregs, MDSCs, and TAMs compared to other cancer cells [101, 102]. Notably, CSCs produce transforming TGF-β, which induces a more differentiated and functional phenotype in Tregs [101, 103]. In return, recruited Tregs secrete vascular endothelial growth factor A (VEGFA), promoting angiogenesis and enhancing CSC stemness, progression, and control [101]. Tregs also contribute to the induction of EMT process in CSCs, fostering their invasive properties. In addition, MDSCs greatly expand in the presence of CSCs and create an immunosuppressive environment, as MDSCs could utilize multiple mechanisms, including arginase and TGF-β, to hinder T cell proliferation and function [101]. Meanwhile, TAMs play a vital role in promoting CSC proliferation and invasion by releasing PDGF, TGF-β, IL-8, and CXCL12, which favor CSC stemness [101, 104]. Moreover, TAMs produce milk-fat globule-epidermal growth factor-VIII (MFG-E8), conferring upon CSCs the ability to promote tumorigenicity and resist anticancer drugs [105]. Upregulation of CD90 were also observed in CSCs from liver cancer, gastric cancer, colon cancer and glioma. This upregulation in CSCs were demonstrated to directly interact with TAMs, reinforcing their stem cell state [106].

### Influence of the TME secretome on CSC-driven tumor progression

IL-6 is primarily secreted by cancer cells and the surrounding TAMs and stromal cells such as CAFs mentioned in the previous section, which induces the development of CSCs, supports their proliferation via JAK/STAT signaling pathway, and facilitates DNA repair mechanisms in response to chemotherapies in CSCs (Fig. 2) [107]. IFN-γ, which traditionally plays a crucial role in T cell recruitment and antigen presentation function of dendritic cells, has recently been implicated in CSC maintenance. IFN-γ produced by cytotoxic T cells accelerates the expansion of chronic myeloid leukemia stem cells, while IFN-γ secreted by CSCs from chemo-resistant tumors upregulates transcription factors promoting M-CSF release and stemness-related properties [108]. TNF-α contributes to EMT in cholangiocarcinoma and renal carcinoma, upregulates the expression of SLUG through the NFκB/HIF1α signaling pathway to maintain breast CSC behavior, and augments CSC characteristics of oral squamous cell carcinoma (OSCC) cells via the Notch-Hes1 pathway [109].

TGF-β, the most common growth factor in the TME, maintains the CSC niche and induces EMT [110]. It is highly enriched in chemo-resistant TNBC cells and their CSC subpopulation, with TGF-β^+^ cells also expressing high levels of CSC markers, such as CD44 and ALDH [111]. Another growth factor, VEGF, is also implicated in promoting CSC stemness, which augments the production of CSC function-related proteins such as Rho family guanosine triphosphatases (GTPases) via the VEGF/NRP signaling pathway [112]. In addition, VEGF facilitates the migration of endothelial cells in the TME, further promoting angiogenesis and metastasis [113].

The cytokine chemokine ligand 12 (CXCL12) and its receptor CXCR4 are strongly associated with EMT and drug-resistance of CSCs. Specifically, CXCR4 is overexpressed in glioblastoma progenitor cells, and in OSCC, CXCL12 secreted from CAFs recruits monocytes with M2 macrophage behaviors, considerably increasing CSC expansion and inhibiting tumor cell apoptosis [114].

### Influence of the hypoxia microenvironment on CSC-driven tumor progression

The hypoxic tumor microenvironment, a common condition in solid tumors, arises due to transiently or constantly disrupted angiogenesis, leading to insufficient oxygen supply to proliferating tumor cells [115]. This condition increases the likelihood of metastasis, worsens prognosis, enhances CSC behavior, and promotes resistance to chemotherapy and immunotherapy [115].

In hypoxic environments, two crucial transcription factors, hypoxia-inducible factors (HIFs), especially HIF-1α and HIF-2α, are stimulated [116]. Required for CSC maintenance and self-renewal, HIFs upregulate the expression of stemness-related genes, including *Oct4*, *Nanog*, and *Sox2*, give rise to abnormal angiogenesis through mediating the secretion of pro-angiogenic factors such as VEGF, and activate DNA repair mechanisms, thereby promoting tumor progression and drug resistance [116, 117]. Besides, the hypoxia environment induces the EMT by enhancing the expression of EMT-promoting genes, such as *SNAI1*, *SNAI2*, *TWIST1*, and *TGFβ1*, which ultimately stimulates the migration of tumor cells to distant tissues [116].

## Cancer stem cells in immune evasion and therapy resistance

In the intricate landscape of the TME, CSCs, though constituting a minor fraction of the tumor population, wield substantial influence over tumor progression, recurrence, and metastasis. Simultaneously, CSCs possess the remarkable capability to elude the host's immune surveillance and engage in dynamic interplay with the TME, thereby conferring resistance to therapeutic regimens. A comprehensive understanding of these intricate interactions assumes pivotal significance in shaping effective therapeutic strategies. In this section, we delve into the nuanced mechanisms employed by CSCs to evade immune surveillance and withstand conventional therapies, establishing a foundational basis for potential approaches designed to surmount these resilient cells.

### Immune checkpoints and immune-activating molecules

The activation of immune cells by tumor cells is a multifaceted and dynamic process that involves intricate interactions within the tumor microenvironment and various constituents of the immune system. Tumor cells possess the capacity to exert both stimulatory and inhibitory influences on immune cells. Notably, tumor cells frequently exhibit the expression of distinctive antigens, encompassing tumor-specific antigens or those arising from mutated proteins. These antigens, discerned as foreign entities by the immune system, can be presented by tumor cells on their surface through the utilization of MHC molecules.

In the context of effective T cell activation, the provision of co-stimulatory signals assumes paramount significance. These signals, frequently conferred by surface molecules such as CD28 and 4-1BB on APCs and other immune cell types, are indispensable for ensuring the full activation and proliferation of T cells. Additionally, for NK cells, the activation process involves the recognition of NK ligands (e.g., CD112, CD155, MICA/B, and ULBP) on tumor cells by NK activating receptors (e.g., NKG2D, NKp30, NKp44, and DNAM-1) [118–120].

Over recent decades, the exploration of cancer immunology has identified a spectrum of inhibitory immunoreceptors, including but not limited to programmed cell death protein 1 (PD-1), cytotoxic T lymphocyte-associated protein 4 (CTLA-4), lymphocyte-activation gene 3 (LAG3), T cell immunoglobulin and mucin domain-containing protein 3 (TIM3), T cell immunoreceptor with immunoglobulin and ITIM domain (TIGIT), and B and T lymphocyte attenuator (BTLA) [121]. These receptors have been denominated as "immune checkpoints" due to their pivotal role as regulators of immune responses. In the context of cancer, tumor cells strategically employ immune checkpoint molecules, exemplified by the PD-1 ligand (PD-L1), as mechanisms to suppress the activity of T cells, consequently facilitating immune evasion. The frequent observation of PD-L1 expression on the surface of tumor cells underscores its interaction with PD-1 receptors on immune cells, thereby inducing a state of exhaustion in these immune effectors, ultimately compromising their functional capacity [122].

### Upregulation of immune checkpoint ligands

Emerging research suggests that CSCs employ immune evasion through the upregulation of immune checkpoint ligands [122–125]. In various types of cancer, including gastric, breast, malignant mesothelioma, bladder, lung, melanoma, and pancreatic cancers, PD-L1 expression is associated with CSC marker expression, tumor growth, and aggressiveness [124]. Moreover, the expression of Cytotoxic T Lymphocyte antigen 4 (CTLA-4) is higher on CSCs compared to normal cancer cells [126]. CTLA-4 acts as an analog of CD28 and suppresses T cell activation by blocking co-stimulation [125]. In the context of lymphoma, elevated CTLA-4 expression is linked to an increased proportion of lymphoma stem cells and enhanced proliferation and invasion of lymphoma cells via the TGF-β pathway [125]. Furthermore, CTLA-4 on CSCs promotes the proliferation of regulatory T cells, which primarily serve to inhibit T cell function [125]. Additionally, the transmembrane protein CD47 plays a crucial role in immune evasion for CSCs [127]. It generates "Don't eat me" signals, which protect self-cells, including CSCs, from being engulfed by macrophages through the SIRPa/CD47 signaling pathway [127]. High expression of CD47 on CSCs in AML, liver, and lung cancers enhances their survival chances, contributing to tumor relapse [127].

### Downregulation of immune-activating molecules

Apart from upregulating immune checkpoint ligands, CSCs also downregulate co-stimulatory molecules, such as CD80, through DNA methylation [124]. The interaction between CD28 on T cells and CD80 on APCs is crucial for providing a co-stimulatory signal that activates T cells [127]. Without this co-stimulation, T cells cannot be fully activated, even when they encounter tumor antigens presented by MHC molecules [127]. Moreover, CSCs exhibit a characteristic reminiscent of embryonic stem cells by downregulating the expression of HLA-I proteins, allowing them to evade recognition and attack by CD8^+^ T cell immunity [128]. On the other hand, breast cancer stem cells (BCSCs) have been observed to decrease the expression of NKG2D ligands, specifically MICA and MICB, through the expression of an oncogenic microRNA called miR20a. This reduction in NKG2D ligands enhances BCSC resistance to NK cell cytotoxicity, leading to increased lung metastasis [129].

### Mechanisms of CSC therapeutic resistance

Dormancy is a state in which cells remain viable but cease to proliferate. In the context of cancer, including CSCs, therapeutic stress can trigger the activation of dormancy mechanisms, enabling these cells to metastasize and evade anti-cancer treatments. Within the realm of CSCs, there exists a distinct subpopulation known as dormancy-competent CSCs, capable of transitioning between periods of dormancy and rapid growth [130]. These cells possess the unique ability to self-renew indefinitely, initiate dormancy in response to treatment, and differentiate into highly proliferative cells, thus contributing to cancer relapse [130]. CSCs also exhibit various adaptive features that enhance their survival under environmental stress. These include increased drug efflux capacity, anti-apoptotic capabilities, and DNA repair proficiency, collectively enabling their prolonged survival [131]. Furthermore, it has been reported that CSCs exhibit lower levels of reactive oxygen species (ROS) compared to their more differentiated counterparts. This characteristic renders CSCs less susceptible to DNA damage caused by radiation exposure [131]. Another significant characteristic associated with CSCs is the acquisition of the EMT phenotype. This process involves morphological changes towards a fibroid appearance, heightened invasiveness characterized by increased motility and tissue infiltration, thus fostering metastasis. Additionally, CSCs undergoing EMT exhibit resistance to apoptosis, evading cell death signals critical for therapeutic-induced cancer cell elimination. Moreover, EMT prompts an upsurge in extracellular matrix components, reshaping the TME to favor growth and progression while potentially impeding therapeutic agent penetration. Previous studies have established a close association between the EMT process and CSCs, promoting a more stemness-like phenotype in cancer cells and consequently augmenting tumor invasion and metastasis [132].

In addition, CSCs exhibit radioresistance, often accompanied by increased EpCAM expression, leading to reduced radiation-induced DNA damage [133]. The elevation in cancer cell stemness following radiation can be attributed to heightened AKT activation, resulting in a hybrid epithelial/mesenchymal phenotype characterized by increased contractility and invasiveness [133, 134]. The consideration of EpCAM-mediated resistance development in radiation therapy may prove valuable for assessing the risk of local recurrence in cancer patients [133].

In summary, CSCs evade the immune system through upregulation of immune checkpoint ligands (e.g., PD-L1, CTLA-4, and CD47) and downregulation of immune-activating molecules (e.g., CD80, HLA-I, and MICA/MICB). CSCs also possess adaptive features and EMT phenotype and can enter dormancy under stress. These distinct characteristics allow them to evade current therapeutic approaches such as chemotherapy, radiotherapy, and T or NK cell-based therapy. Understanding these mechanisms is vital for developing targeted therapies to overcome CSC-mediated resistance and improve cancer treatments.

## Therapeutic implications of cancer stem cells

**Fig. 3:**
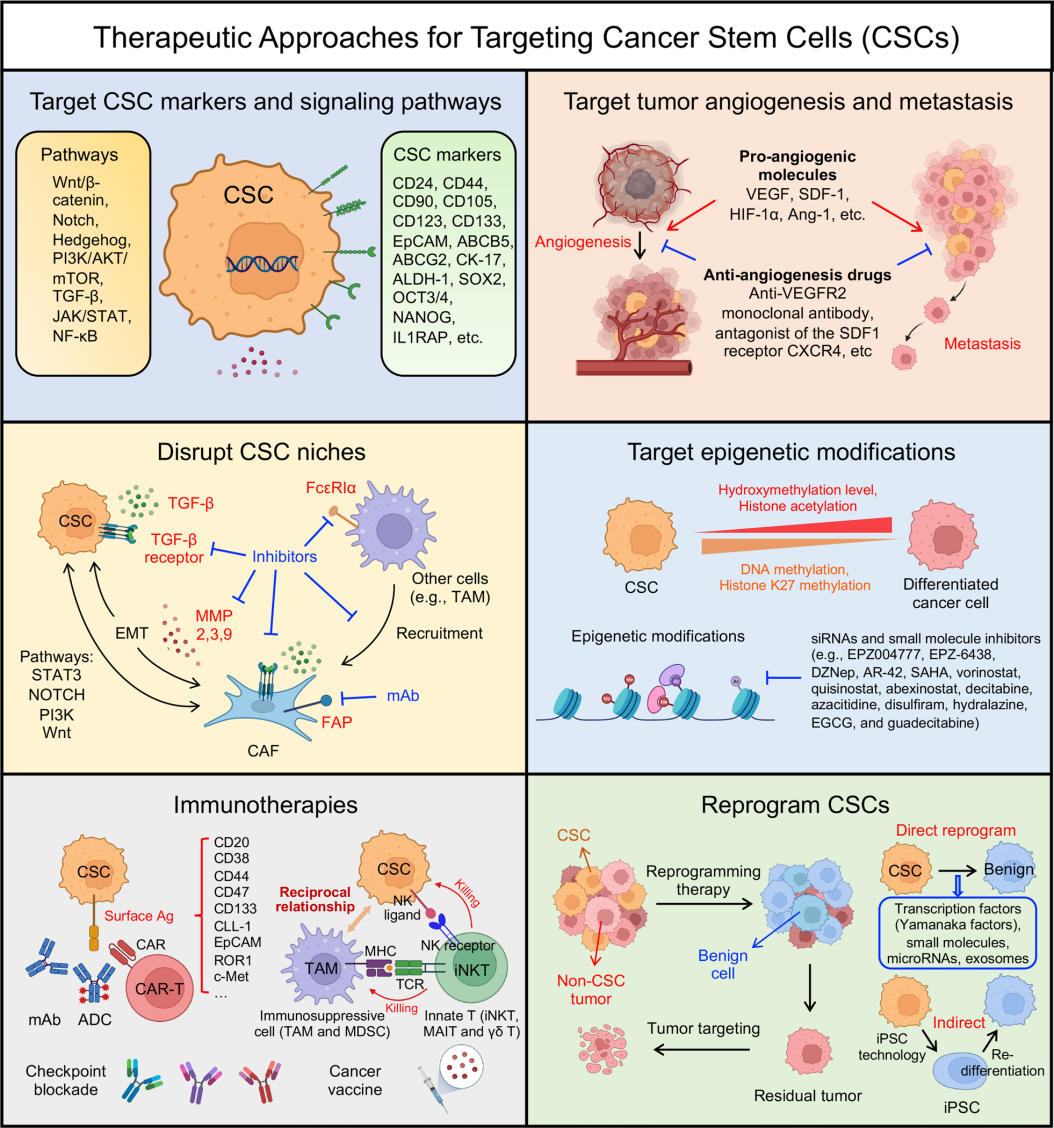
Therapeutic approaches for targeting CSCs. The figure outlines six distinct strategies to target CSCs, including targeting CSC markers and signaling pathways, targeting CSC-associated tumor angiogenesis and metastasis, disrupting CSC niches, targeting epigenetic modifications, exploring immunotherapies, and reprogramming CSCs. VEGF, vascular endothelial growth factor; SDF-1, stromal cell-derived factor-1; HIF-1α, hypoxia-inducible factor 1-alpha; Ang-1, angiopoietin-1; TGF-β, transforming growth factor-beta; EMT, epithelial-mesenchymal transition; FAP, fibroblast activation protein; mAb, monoclonal antibody; ADC, antibody drug conjugate; TAM, tumor-associated macrophage; MDSC, myeloid-derived suppressor cell

In light of CSCs' critical role in tumor relapse, metastasis, and therapy resistance due to their unique biological properties and complex interactions with immune cells and the tumor microenvironment, the development of targeted therapies against CSCs has become imperative. Common strategies involve focusing on CSC-associated markers (e.g., CD20, CD52, CD44v6, CD123, and EpCAM) using monoclonal antibodies and chimeric antigen receptor (CAR)-engineered T (CAR-T) cells to target CSC markers [38, 135–140]. The marker ALDH peptide is utilized in dendritic cell-based vaccines against CSCs through synthetic high-density lipoprotein nanodiscs [141]. Another potential target is the signaling pathways associated with CSCs (e.g., Wnt, Notch, JAK-STAT, PI3K, and NF-κB pathways), which are crucial for CSC survival and persistence [24]. The use of monoclonal antibodies against pathway ligands, along with inhibitors like γ-secretase inhibitors, smoothened (SMO) inhibitors, β-catenin inhibitors, and BCL-2 inhibitors, serves to disrupt these pathways, leading to CSC apoptosis and the disruption of interactions between CSCs and other immune cells [142–145]. In this section, we will explore various strategies for targeting CSCs, shedding light on their potential significance in cancer treatment (Fig. 3).

### Target CSC-specific markers and signaling pathways

A range of cell markers has been employed for the characterization and isolation of cell subsets enriched with CSCs. These markers include CD24 [146], CD44 [146–150], CD90 [151, 152], CD105 [153, 154], CD123 [155–157], CD133 [158–163], epithelial cell adhesion molecule (EpCAM) [149], ATP-binding cassette B5 (ABCB5) [164], ATP-binding cassette G2 (ABCG2) [163, 165], cytokeratin 17 (CK-17) [166], Integrin α7 [167], Interleukin-1 receptor accessory protein (IL1RAP) [168, 169], aldehyde dehydrogenase 1 (ALDH-1) [170, 171], SRY-box 2 (SOX2) [171–173], octamer-binding transcription factor 3/4 (OCT3/4) [166, 172, 173], NANOG [166, 172, 173], among others. However, it is important to note that there is currently no universally standardized and accurate marker for identifying CSCs, and the specific CSC markers may vary across different cancer types [123]. The identification and targeting of surface markers specific to CSCs hold significant potential for selectively eliminating CSC populations, thereby interrupting tumor growth and preventing disease relapse.

Various combinations of drugs and antibodies have demonstrated successful targeting of CSC surface markers across different cancer types (Fig. 3) [174]. Notably, an anti-CD133-drug conjugate exhibited significant suppression of hepatocellular and gastric cancers [175], while polymeric nanoparticles targeting CD133 exhibited tumor-killing effects in breast cancer [176]. Treatment with anti-CD44 demonstrated inhibition of tumor proliferation and increased apoptosis in acute myeloid leukemia (AML) [177]. Clinical trials employing anti-EpCAM have shown tumor suppression in colon and prostate cancer [178, 179], and an anti-CD123 scFv-pseudomonas exotoxin exhibited cytotoxic effects on AML and chronic myeloid leukemia (CML) tumor cells [180]. Additionally, an anti-CD24 monoclonal antibody inhibited the growth of colorectal and pancreatic cancer tumors in vivo [181]. These findings highlight the potential of targeted therapies utilizing specific CSC surface markers for effective cancer treatment.

Numerous signaling pathways that play critical roles in the survival, proliferation, self-renewal, and differentiation processes of normal stem cells undergo dysregulated activation or repression during tumorigenesis or in the context of CSCs. The intricate interplay of endogenous and exogenous genes, as well as microRNAs, tightly regulates these complex pathways [24]. Consequently, these signaling pathways exert influence over the expression of downstream genes in CSCs, including cytokines, growth factors, and genes associated with apoptosis, antiapoptosis, proliferation, and metastasis [24]. The Wnt/β-catenin pathway is frequently disrupted in CSCs and plays a vital role in facilitating the self-renewal, maintenance, and initiation of tumors [182–186]. The Notch pathway is involved in regulating cell fate decisions and self-renewal processes in CSCs, and aberrant activation of the Notch pathway can contribute to the persistent maintenance of CSCs and promote the progression of tumors [187–191]. The Hedgehog pathway, when abnormally activated, is associated with CSC characteristics such as self-renewal, survival, and resistance to therapeutic interventions, and inhibiting this pathway has shown potential in reducing CSC populations and impeding tumor growth [192–195]. The PI3K/AKT/mTOR pathway regulates multiple cellular processes and its dysregulation in CSCs promotes self-renewal, survival, and resistance to therapy, and targeting this pathway holds promise in inhibiting CSC activity and enhancing the effectiveness of therapeutic approaches [196–199]. The TGF-β pathway exerts complex effects on CSCs, influencing their self-renewal, differentiation, and interactions with the tumor microenvironment, and dysregulation of the TGF-β pathway can contribute to the persistent maintenance of CSCs and the progression of tumors [200–202]. The JAK/STAT pathway is involved in the maintenance, proliferation, and immune evasion of CSCs, and dysregulated JAK/STAT signaling in CSCs can promote tumor growth and resistance to therapy [61, 203–206]. The NF-κB pathway is associated with the survival, self-renewal, and resistance to apoptosis of CSCs, and dysregulation of the NF-κB pathway can enhance CSC properties and contribute to tumor progression and therapy resistance [207–210]. The abnormal GPCR signaling profoundly influences CSCs within the ECM, particularly through YAP/TAZ activation. GPCR activation initiates cascading events, modulating YAP/TAZ phosphorylation, enhancing the stem-like properties and therapy resistance of CSCs [100].

Various agents have been explored to target the CSC pathways and shown promising suppression or depletion of CSCs. Numerous pharmacological agents targeting the Wnt signaling pathway have undergone assessment either through clinical trials or preclinical testing, including Ipafricept (OMP-54f28/FZD8-Fc), PRI-724, LGK974, ETC-159, OMP-18R5, CWP232228, CWP232291, NCB-0846, anti-Frizzled-6, and anti-Frizzled-5 [24, 174, 211–216]. Pharmacological agents targeting the Notch signaling pathway as inhibitors include RO4929097, DAPT, MK-0752, PF-03084014, BMS-906024, BMS-986115, CB-103, LY3039478, LY900009, and the DLL4-targeting antibody Demcizumab [142, 217–224]. In addition, Nitidine chloride, Gemcitabine, Vismodegib, Sonidegib, Taladegib, and Glasdegib have been associated with the inhibition of the Hedgehog signaling pathway [225–230]. The utilization of Napabucasin and Ruxolitinib to inhibit the JAK/STAT3 pathway has been demonstrated to reduce the viability of hematopoietic and prostate CSCs and attenuate their tumorigenic properties [61, 231, 232]. Overall, these findings highlight the potential of diverse pharmacological agents targeting CSC pathways, paving the way for novel and promising approaches to suppress or deplete CSCs and advance cancer therapeutics.

### Target CSC-associated tumor angiogenesis and metastasis

Tumor angiogenesis poses another challenge in combating tumor and CSCs. CSCs play a role in this process through vasculogenic mimicry (VM), where they transform into endothelial-like cells, forming tube-like structures without true endothelial cells [233]. Hypoxia in the tumor environment activates various signaling pathways (e.g., HIF, c-Myc, Sox-2, Oct-4, adenosine/STAT3/IL-6 pathway, MAPK/ERK pathway, Notch, Wnt, Hedgehog, and Hippo signaling) [234], promoting the multi-directional differentiation potential of CSCs and influencing CSC transformation into incomplete endothelial cells that exhibit VM in different tumor types (e.g., oral squamous cell carcinoma, breast cancer, liver cancer, colorectal cancer, and cholangiocarcinoma) [235–239].

The intricate interplay between CSCs and immune cells notably impacts VM and tumor angiogenesis, with endothelial progenitor cells (EPCs) playing a crucial role. EPCs, possessing the ability to differentiate into endothelial-like cells, actively participate in the formation of vascular-like structures alongside tumor cells [240]. While the exact mechanisms remain incompletely understood, studies have confirmed the reciprocal interactions between CSCs and EPCs. Notably, CD133^+^ CSCs in glioblastoma promote tumor angiogenesis by secreting pro-angiogenic factors such as VEGF and stromal-derived factor-1 (SDF-1), attracting EPCs to the tumor site [241]. Similarly, in colon cancer, CSCs secrete VEGF, HIF-1α, and angiopoietin-1 (Ang-1), facilitating the recruitment and migration of EPCs [242]. Subsequently, migrated EPCs reciprocate by secreting more VEGF, further augmenting CSCs to acquire EPC-like characteristics and contributing to tumor angiogenesis [243]. Single-cell RNA sequencing analysis in colorectal cancer provides additional evidence of the close association between CSCs and EPCs, offering valuable insights into their interactions [244].

The significance of pro-angiogenic molecules in these processes has led to the development of anti-angiogenesis drugs like bevacizumab, which specifically inhibits VEGF secreted by CSCs or other vascular-niche cells, disrupting the signaling pathway that stimulates angiogenesis (Fig. 3). Preclinical studies in brain tumor-bearing xenograft mice have demonstrated its efficacy [245, 246]. Additionally, anti-VEGFR2 monoclonal antibody (DC101) and the antagonist of the SDF1 receptor CXCR4 (AMD3100) have also shown significant reduction in microvessel density in CSC-high tumor-bearing mice [241]. However, the clinical translation of these therapies has faced challenges, with patients exhibiting resistance to these treatments [247, 248], possibly due to the emergence of VEGF-independent angiogenesis pathways or other redundant mechanisms in the tumors [249]. Hence, to improve therapeutic approaches targeting tumor angiogenesis and prevent metastasis, it is crucial to gain a deeper understanding of the intricate interactions between cells involved in VM and angiogenesis, including the complex interplay between CSCs and EPCs mentioned previously. By elucidating the elusive mechanism of CSC and EPC interactions, we may unlock new opportunities for more effective strategies in the ongoing fight against cancer.

### Disrupt CSC niches

The complex microenvironment of the CSC niche plays a critical role in CSC self-renewal and tumor progression, comprising various components such as CSCs, non-CSC tumor cells, stromal cells, ECM proteins, and signaling molecules. Targeting key components and pathways within the CSC niche holds promise for developing effective therapies. A crucial player in this microenvironment is CAFs, which secrete niche factors (e.g., VEGF, CXCL12, PDGF, and HGF) and activate Wnt and NOTCH pathways, remodel the ECM, and influence CSC stemness and immune responses. An enticing strategy involves targeting CSC-stimulating CAFs expressing α-SMA and fibroblast activation protein (FAP). Although some side-effects like enhanced tumor hypoxia and Treg infiltration may arise with α-SMA targeting [250], FAP-targeting strategies show significant antitumor efficacy in preclinical models of lung, colon and pancreatic cancers (Fig. 3). Preclinical investigations have explored various approaches, including FAP deletion through LacZ knock-in or Diphtheria toxin targeting FAP^+^ CAFs in lung cancer, colon cancer, and pancreatic cancer xenograft models, demonstrating notable antitumor efficacy [251, 252]. Additionally, maytansinoid-conjugated antibody (FAP5-DM1), toxin-conjugated antibody (αFAP-PE38), and FAP inhibitors (e.g., PT630 and PT100) have shown effective FAP-targeted killing in breast cancer and melanoma [253, 254]. Clinical studies with the antibody inhibitor sibrotuzumab exhibited both safety and potential for tumor inhibition in lung and colon cancer [251, 255]. However, a challenge associated with this strategy is the limited penetration of large-molecule therapeutics, such as antibodies, into desmoplastic tissues within the tumor microenvironment [256]. Addressing this limitation could pave the way for more efficient and successful FAP-targeted therapies in the future.

Disrupting the crosstalk between CAFs and CSCs, along with related pathways, represents another promising therapeutic avenue. The STAT3 signaling pathway, activated by IL6/IL6R, plays a pivotal role in the CSC niche by contributing to increased MMP secretion and sustaining the CSC-stimulating phenotype. Preclinical studies have demonstrated the efficacy of STAT3 pathway inhibitors, such as Stattic [257], C188-9 [258], OPB-31121 [259], OPB-51602 [260], AZD9150 [261], and STAT3 decoy oligonucleotide [262], as well as drugs targeting IL6/IL6R, including Siltuximab [263], Tocilizumab [264], and Olamkicept [265], across various tumor models. Furthermore, the activation of the NOTCH pathway through CXCR2 secretion from the STAT3 pathway has prompted investigations into CCL2 neutralizing antibodies (e.g., Carlumab and PF-04136309) [266] and NOTCH inhibitors like DAPT [267], INCB3619 [268], and Crenigacestat [269]. The TGF-β pathway, secreted by CAFs and mediated by autocrine signaling loops involving TGF-β and SDF-1, contributes to tumor stiffness and desmoplasia in breast and gastric cancer. Targeting this pathway includes inhibiting SDF-1 and CXCR4 with Plerixafor [270], neutralizing TGF-β receptors with antibodies like GC1008 [271] and LY3022859 [272], or using inhibitors like Ki26894 [273], LY2109761 [274], and PF-03446962 [275]. Downstream components of the TGF-β pathway, including PI3K inhibitors like BKM120 [276] or Ly294002 [277], have shown antitumor efficacy in preclinical trials, while clinical trials with PX-866 [278], alpelisib [279], PQR309 [280], and pictilisib [281] have demonstrated promising antitumor activity in patients with solid tumors. Additionally, CAF-secreted MMP2, MMP3, and MMP9 remodel the ECM, promote EMT [282], enhance the expression of CSC-related markers through the Wnt pathway, and worsen therapeutic resistance. Inhibiting MMPs, such as Ilomastat [283], has effectively suppressed the conversion of CSC phenotype from tumor cells in animal models.

Apart from targeting signaling pathways, disrupting interactions between different cell types within the CSC niche holds great potential. For instance, in squamous cell carcinoma (SCC), targeting CSC-derived IL-33-induced FcεRIα^+^ TAM differentiation could destabilize CSCs and enhance cancer treatment outcomes [284]. Furthermore, as discussed in the previous section, understanding and targeting the interactions between cells in the vascular niche, another component of the CSC niche, may provide valuable insights for further therapeutic advancements.

### Target epigenetic modifications in CSCs

The emergence of CSCs has been linked to abnormal epigenetic alterations in normal cells, leading to sustained primed epigenetic modifications that perpetuate aberrant differentiation and tumorigenesis, even after the oncogene’s cessation [285]. Notably, histone methylation, a crucial epigenetic change regulated by histone methyltransferases (HMTs) and histone demethylases (HDMs), plays a pivotal role in maintaining normal cellular functions and development. Dysregulation of histone methylation, exemplified by elevated H3K27me3 and reduced H3K4me2 levels in triple-negative breast cancer stem cells, sustains their stem cell-like properties and tumorigenic potential [286]. Targeting histone methyltransferase DOT1L through siRNAs has demonstrated promise in attenuating tumor invasion and enhancing chemosensitivity (Fig. 3) [287]. Small molecule inhibitors such as EPZ004777 showed selective blockade of H3K79 methylation and suppression of the expression of leukemogenic genes in vitro, as well as DOT1L inhibition and extended survival against mixed lineage lymphoma in vivo [288]. Similarly, the methyltransferase EZH2, which induces H3K27me3 and represses tumor suppressor genes, is a potential therapeutic target with inhibitors like EPZ-6438 and 3-deazaneplanocin-A (DZNep) [289].

Moreover, aberrant histone acetylation in CSCs presents another therapeutic avenue. Histone deacetylases (HDACs), including HDAC1, HDAC7, and HDAC11, are frequently overexpressed in CSCs, contributing to their stem cell-like properties [287]. Inhibition of HDACs, particularly HDAC11, has shown promise in reducing the expression of stem cell markers and enhancing treatment sensitivity in certain cancer types. HDAC inhibitors (HDACis) AR-42, SAHA [290], vorinostat [291], quisinostat [292], and abexinostat [293] have demonstrated efficacy in targeting CSCs and improving cancer treatment outcomes [294]. For instance, entinostat has been observed to diminish the expression of CSC markers in triple-negative breast cancer and shown the potential to enhance the sensitivity of pancreatic cancer cells to gemcitabine by upregulating miR-203 expression [295]. These findings suggest that modulating histone acetylation holds potential as a strategy to regulate CSCs and enhance the effectiveness of cancer therapies.

Additionally, DNA methylation, the addition of a methyl group to cytosine bases in DNA, contributes to gene silencing and is a critical aspect of epigenetic modification. DNA methyltransferases (DNMTs), including DNMT1, DNMT3A, and DNMT3B, play key roles in DNA methylation regulation. DNMT1 maintains DNA methylation patterns during cell division, and targeting DNMT1 has shown promise in inducing apoptosis and reducing tumorigenesis in lung CSCs [296]. Inhibitors of DNMTs, such as decitabine, azacitidine, disulfiram, hydralazine, and EGCG, have demonstrated efficacy in various cancers, including AML, MDS, lung, and colorectal cancer [297–299]. Moreover, mutations in DNMT3A and DNMT3B have been associated with CSCs' undifferentiated state and the activation of proliferation pathways, contributing to treatment resistance. Inhibitors of DNMT3s, such as guadecitabine (SGI-110) and SGI-1027, exhibit potent antitumor activity against lymphomas and prostate cancer [300, 301]. Targeting epigenetic modifications, including histone methylation, histone acetylation, and DNA methylation, presents promising strategies to subvert tumor metastasis and improve the efficacy of cancer treatments in CSCs. Epigenetic-based therapies hold great potential in the battle against cancer and may significantly impact clinical outcomes.

### Immunotherapies

Harnessing the immune system to target CSCs represents a promising avenue in cancer therapeutics. Immune-based approaches, such as adoptive cell therapy, immune checkpoint inhibitors, and cancer vaccines, have emerged as effective strategies to enhance the immune response against CSCs and potentially improve treatment outcomes (Fig. 3).

Several cell surface proteins serve as CSC markers, including CD20, CD38, CD44, CD47, CD117, CD123, CD133, CD166, CD171, CLL-1, EpCAM, ROR1, and c-Met. These markers present potential targets for CAR-T cell therapy and novel monoclonal antibodies, offering opportunities to specifically engage CSCs [38]. By redirecting the immune system to recognize CSC-specific antigens, CAR-T cell therapy holds promise in eradicating CSCs and preventing tumor relapse. However, current CAR-T cell therapy faces limitations in treating CSCs. These challenges encompass shared antigens between CSCs and normal adult stem cells, antigenic heterogeneity among CSCs, inadequate persistence and trafficking of CAR-T cells to the CSC microenvironment, and the presence of diverse cellular and acellular immunosuppressive factors [38]. Addressing these limitations will be essential to enhance the efficacy and safety of CAR-T cell therapy against CSCs. Exploring alternative strategies, such as combinatorial approaches with other immunotherapies or engineering CAR-T cells to better navigate the CSC microenvironment, may offer potential solutions to overcome these obstacles.

Apart from CAR-T cells, other immune cell types, including natural killer (NK) cells and innate T cells such as invariant natural killer T (iNKT), gamma-delta T (γδ T), and mucosal-associated invariant T (MAIT) cells, hold significant potential for treating CSCs. These cells express high levels of NK activating receptors like NKG2D, DNAM-1, and NKP30, enabling them to recognize and eliminate CSCs through NK-mediated pathways [302–305]. Furthermore, these innate cells can be genetically engineered with CARs to augment their anti-CSC capabilities [306–308]. In addition to their direct targeting of CSCs, innate cells have demonstrated the ability to modulate the TME by interacting with TAMs and MDSCs [12, 15, 16, 309]. For instance, in pro-inflammatory environments, iNKT cells can recognize TAMs and MDSCs through CD1d/iNKT TCR recognition, potentially influencing the TME [310–313]. Harnessing NK and innate T cell-based therapies offers a promising approach to simultaneously target CSCs and modulate the TME. Exploiting the inherent properties of these cells, combined with genetic engineering through CAR technology, presents a multifaceted strategy with the potential to improve cancer treatment outcomes by addressing the complexities of both CSCs and the TME.

Given the observed upregulation of checkpoint pathways on CSCs, these cells can evade immune surveillance. Inhibiting these checkpoint pathways can reinvigorate immune cells and foster a more potent and enduring anti-tumor response, effectively targeting CSCs [36]. Additionally, cancer vaccines represent an alternative strategy for CSC targeting. By presenting CSC-specific antigens to immune cells, particularly dendritic cells (DCs), cancer vaccines can instruct the immune system to recognize and eliminate CSCs, thereby eliciting more sustained and durable immune responses [314].

The combination of these immune-based approaches holds great promise in tackling the challenges associated with CSCs, including their therapeutic resistance and potential role in tumor relapse. Moreover, the targeting of CSCs through immune-mediated mechanisms may complement conventional treatments like chemotherapy and radiation therapy, leading to synergistic effects and improved overall treatment outcomes. However, it is crucial to continue research efforts to better understand the complexity of CSC-immune interactions, optimize treatment strategies, and develop innovative combination therapies that exploit the full potential of the immune system to combat CSC-driven tumor growth and progression.

### Reprogram CSCs

Reprogramming CSCs by inducing their differentiation or conversion into a non-tumorigenic state holds significant promise in cancer therapeutics [315]. Such reprogramming strategies aim to disrupt the characteristic properties of CSCs, including their self-renewal capabilities and therapy resistance, with the ultimate goal of attenuating tumor growth and improving treatment outcomes (Fig. 3).

CSCs possess the capability to undergo differentiation, a process by which they mature into non-stem cell progeny with limited tumorigenic potential. Therapeutic interventions aimed at promoting CSC differentiation hold promise in halting their self-renewal and impeding tumor regeneration. Crucial signaling pathways, including Notch, Wnt, and Hedgehog, play key roles in maintaining CSC properties and represent viable targets for inducing differentiation. Disrupting these pathways can coerce CSCs to relinquish their stem cell-like features, rendering them more susceptible to conventional cancer treatments. Furthermore, reprogramming strategies aim to convert CSCs into non-tumorigenic cell types, reducing their ability to drive tumor growth and resist therapy [315]. Cellular reprogramming techniques, like induced pluripotent stem cell (iPSC) technology, have demonstrated potential in transforming CSCs into non-tumorigenic cells through alterations in epigenetic and transcriptional profiles [316, 317]. Diverse approaches have been explored, such as delivering transcription factors (e.g., Yamanaka factors), small molecules, microRNAs, and exosomes in combination [318–323]. This reprogramming can potentially replace CSCs and hinder tumor progression. Additionally, targeting epigenetic regulators, such as DNA methyltransferases and histone deacetylases, can promote CSC self-renewal and therapy resistance. Inhibiting these regulators through epigenetic therapies may alter the CSC phenotype, sensitizing them to standard cancer treatments [324].

In conclusion, reprogramming CSCs through differentiation induction or conversion to a non-tumorigenic state represents a promising frontier in cancer research. By disrupting the core characteristics of CSCs, these approaches offer new possibilities for effectively eliminating CSC-driven tumorigenesis, enhancing treatment responses, and ultimately improving the prognosis for cancer patients. Continued research and innovative strategies are essential to further unravel the complexities of CSC biology and translate reprogramming approaches into effective clinical interventions.

## Conclusion

CSCs have been recognized for their heterogeneity and plasticity, which contribute to immune evasion and therapy resistance. As a result, advanced therapeutic strategies have emerged to target these critical cell populations. Recent research has increasingly focused on understanding the dynamic interplay between CSCs and the TME, where both CSCs and immunosuppressive cells in the TME benefit from this symbiotic relationship. Investigating the intricate connection between CSCs and the TME and developing innovative technologies to disrupt CSC niches and impair the interaction between CSCs and the TME hold great promise for improving cancer treatment. This review aims to systematically explore the relationship between CSCs and the TME, shedding light on how the TME aids CSCs in evading immune targeting and promoting therapy resistance. Additionally, we provide a comprehensive summary of the current therapeutic approaches aimed at targeting both CSCs and the TME.

Notwithstanding the progress in developing strategies to target CSCs, several challenges still impede their effective implementation. Firstly, the precise characterization of CSCs in specific tumor types remains incomplete, and there is a lack of consensus on definitive and accurate markers for identifying CSCs [325]. Secondly, current research on CSCs often relies on organoid or humanized mouse models, which fail to fully replicate the complex interactions between CSCs and the TME due to the absence of a native microenvironment. Consequently, these models may not fully capture the intricacies of CSC behavior in the actual tumor context [326]. Thirdly, the environmental factors governing CSC niches are not yet thoroughly elucidated, hindering the comprehensive understanding of CSC biology [24, 327]. Addressing these challenges is crucial to advancing our knowledge of CSCs and their interactions with the TME, thus paving the way for more effective CSC-targeted therapeutic approaches.

## Data Availability

All data generated or analyzed during this study are included in this published article.

## Abbreviations

CSC: Cancer stem cell
TME: Tumor microenvironment
TAMs: Tumor-associated macrophages
MDSCs: Myeloid-derived suppressor cells
CAFs: Cancer-associated fibroblasts
Tregs: Regulatory T cells
EMT: Epithelial-to-mesenchymal transition
CAR-T: Chimeric antigen receptor (CAR)-engineered T
SMO: Smoothened
PD-1: Programmed death-1
PD-L1: PD-1 ligand
CTLA-4: Cytotoxic T lymphocyte antigen 4
LAG-3: Lymphocyte-activation gene 3
TIM-3: T cell immunoglobulin and mucin domain-containing protein 3
TIGIT: T cell immunoreceptor with immunoglobulin and ITIM domain
BTLA: B and T lymphocyte attenuator
ROS: Reactive oxygen species
MSCs: Mesenchymal stem cells
ECM: Extracellular matrix
HA: Hyaluronan
VEGF: Vascular endothelial growth factor
bFGF: Basic fibroblast growth factors
TNBC: Triple-negative breast cancer
GSCs: Glioblastoma stem cells
PDAC: Pancreatic ductal adenocarcinoma
VEGFA: Vascular endothelial growth factor A
GTPases: Guanosine triphosphatases
CXCL12: Cytokine chemokine ligand 12
ABCG2: ATP-binding cassette G2
EpCAM: Epithelial cell adhesion molecule
ABCB5: ATP-binding cassette B5
CK-17: Cytokeratin 17
OCT3/4: Octamer-binding transcription factor 3/4
SOX2: SRY-box 2
ALDH-1: Aldehyde dehydrogenase 1
IL1RAP: Interleukin-1 receptor accessory protein
AML: Acute myeloid leukemia
CML: Chronic myeloid leukemia
VM: Vasculogenic mimicry
EPCs: Endothelial progenitor cells
SDF-1: Stromal-derived factor-1
Ang-1: Angiopoietin-1
FAP: Fibroblast activation protein
HDACs: Histone deacetylases
HDACis: HDAC inhibitors
DNMTs: DNA methyltransferases
iNKT: Invariant natural killer T
γδ T: Gamma-delta T
MAIT: Mucosal-associated invariant T
iPSC: Induced pluripotent stem cell
